# Psychological Distress Mediates the Relationship Between Perceived Social Isolation and Medical vs. Recreational Marijuana Use Among Adults in the United States

**DOI:** 10.3390/psychiatryint7020055

**Published:** 2026-03-04

**Authors:** Derek S. Falk, Christian E. Vazquez, Swasati Handique

**Affiliations:** 1 School of Social Work, The University of Texas at Arlington, Arlington, TX 76019, USA; 2 Patient and Community Engaged Research (PACER) Center, Baylor Scott & White Research Institute, Dallas, TX 75204, USA; 3 Department of Family Medicine, Baylor College of Medicine, Temple, TX 76798, USA

**Keywords:** medical marijuana, recreational marijuana, cannabis, psychological distress, perceived social isolation, loneliness, mediation analysis, coping motives

## Abstract

Marijuana use in the United States (U.S.) has diversified alongside expanding legalization, yet little is known about the psychosocial factors that distinguish medical from recreational use. This study examined whether psychological distress mediates the association between perceived social isolation (i.e., loneliness) and marijuana use type among U.S. adults. We analyzed cross-sectional, nationally representative data from the 2024 Health Information National Trends Survey (HINTS, cycle 7). Marijuana use was categorized as medical (including medical and both medical/recreational) versus recreational. Perceived social isolation was measured using the Patient-Reported Outcomes Measurement Information System (PROMIS) Social Isolation t-score, and psychological distress was assessed with the Personal Health Questionnaire (PHQ)-4. Survey-weighted descriptive analyses and a weighted structural equation mediation model accounting for the complex sampling design were conducted. Medical marijuana users reported significantly higher levels of psychological distress and perceived social isolation than recreational users. Greater social isolation was strongly associated with higher psychological distress, and higher distress was associated with a greater likelihood of medical (vs. recreational) marijuana use. The indirect effect of social isolation on marijuana use type through psychological distress was statistically significant, while the direct effect of social isolation was not significant after accounting for distress. Overall, greater perceived social isolation predicted medical marijuana use primarily through elevated psychological distress. These findings suggest that medical marijuana use among U.S. adults may reflect coping with psychological distress linked to social disconnection, underscoring the importance of integrating mental health and social context into clinical and public health approaches to cannabis use.

## 1. Introduction

Marijuana use in the United States (U.S.) has undergone dramatic transformation over the past several decades, shaped by shifting cultural norms, expanding legalization, and growing interest in potential therapeutic benefits. Population-level data show that cannabis consumption has risen steadily since the late 1970s, affecting youth, adults, and older populations alike [1–3]. As legal access has widened, marijuana-related outcomes have also diversified, ranging from increased emergency department visits for cannabinoid hyperemesis syndrome [4,5] to a rising prevalence of marijuana-related disorders, particularly among older adults and Medicare beneficiaries [6,7]. These trends have unfolded against a rapidly evolving policy landscape in which states adopt medical and recreational marijuana laws at different paces, generating heterogeneous patterns of health-related behaviors, such as frequency and mode of cannabis use, polysubstance use (e.g., alcohol simultaneous and co-use), and help-seeking for mental health or substance use concerns as well as downstream health consequences. These patterns vary sociodemographically, with differences observed by age, sex, race/ethnicity, socioeconomic status, and sexual orientation, reflecting unequal exposure, access, and vulnerability across diverse populations [8–10].

One of the most notable developments within this shifting landscape is the growth of medical marijuana use. Medical cannabis users, particularly those who are licensed, tend to be distinguished from recreational users by their health-related motives, more regulated patterns of use, and greater integration into medical systems, whereas recreational users primarily use cannabis for pleasure and social reasons, with unlicensed “medical” users often falling somewhere in between these groups [11]. Recent national data show substantial increases in medical marijuana registrations, changes in authorizing clinicians, and evolving reasons for certification, particularly between 2020 and 2022 [12]. Several studies have documented how medical marijuana is used to manage pain, improve quality of life, and serve as an adjunct to conventional care [13,14], leading to increased interest among both patients and healthcare providers. This expansion has occurred alongside evolving perceptions of risk and benefit, particularly among older adults, who represent one of the fastest-growing groups of marijuana users [15,16]. Research has also examined the broader social and behavioral effects of marijuana policies, including associations with crime [17], traffic fatalities [8], mental health [18,19], and emerging psychoactive markets such as hemp-derived cannabinoids [20]. Youth and young adult patterns have also shifted, with measurable changes in coping-motivated marijuana use [21] and evolving co-use of tobacco products in states with differing policy environments [9].

Loneliness and social isolation are increasingly recognized as important psychosocial correlates of marijuana use across the life course. Qualitative and mixed-methods research suggests that cannabis is often used to cope with feelings of disconnection, psychological distress, and stress, particularly in contexts marked by limited social support or stigma surrounding both mental health and cannabis use [22–24]. Among young adults, marijuana use has traditionally been embedded in social contexts; however, disruptions to social networks, particularly during the COVID-19 pandemic, have been associated with shifts toward more solitary use, which may exacerbate loneliness rather than mitigate it [25–27]. At the population level, increasing normalization of cannabis use over time may further blur distinctions between recreational, social, and self-medicative use, particularly during periods of heightened social stress [28–30].

Emerging evidence also highlights loneliness as a salient factor in marijuana use among older adults, a population historically underrepresented in cannabis research. National survey data from Canada collected during the COVID-19 pandemic demonstrated a significant association between loneliness and cannabis use among older adults, suggesting that cannabis may function as a coping strategy for social isolation later in life [31]. Across age groups, loneliness appears to act both as a risk factor for increased use and as a contextual driver of self-medication motives, particularly among individuals experiencing psychological distress or hazardous cannabis use patterns [23,24]. These findings underscore the importance of incorporating social connection and loneliness into prevention and intervention frameworks addressing marijuana use, especially during periods of social disruption.

Much remains unknown about the psychosocial factors that differentiate medical from recreational cannabis use in the general adult population despite this rich and rapidly expanding evidence base. Prior work has highlighted demographic correlates of cannabis use disorders among medical cardholders [32], the role of political polarization in the destigmatization of medical cannabis markets [33], and long-term research trends across biopsychosocial domains [34]. Relatively few studies have examined how social and psychological experiences shape individuals’ reasons for using marijuana, particularly within nationally representative samples. While mental health and coping motivations have been increasingly recognized as important drivers of marijuana use, especially among individuals experiencing serious psychological distress or social stressors [21,35], little research has directly compared how social isolation and psychological distress relate to medical versus recreational marijuana use. Even less is known about whether psychological distress may function as a mechanism linking social isolation to decisions about marijuana use type, despite theoretical and clinical relevance. In particular, the extent to which perceived social isolation influences marijuana use type and whether this association is explained by underlying psychological distress remains understudied in population-level research.

### 1.1. Theoretical Framework and Hypothesis Development

The present study is grounded in an integrated framework that combines the Self-Medication Hypothesis with the Well-being Optimization Theory (WOT). The link between distress and substance use is viewed through the lens of self-medication, which posits that individuals use psychoactive substances to alleviate specific states of psychological pain or “ego-deficits” [36]. In this context, social isolation acts as a primary stressor that generates the painful affective states (anxiety and depression) that cannabis is then used to “medicate”. While self-medication focuses on the reduction of negatives, the WOT suggests that individuals engage in health behaviors to optimize their overall “wellbeing equilibrium” [37]. According to the WOT, when an individual’s social resources are depleted (social isolation), they experience a “wellbeing deficit”. The shift toward medical rather than recreational categorization represents a cognitive and behavioral transition: the user is no longer seeking “pleasure” (recreational), but is instead attempting to “optimize” a baseline level of functioning that has been compromised by distress. This integrated model suggests that psychological distress is the “signal” that one’s well-being is suboptimal. Consequently, the individual adopts a “medical” framework for marijuana use as a structured attempt at restorative health management.

### 1.2. Hypotheses

Based on this integrated framework, we propose the following hypotheses to be tested using structural equation modeling:

**Hypothesis 1 (Path A)**: *Greater perceived social isolation will be positively associated with higher levels of psychological distress*.
**Hypothesis 2 (Path B):** *Higher psychological distress will be positively associated with an increased likelihood of reporting medical (vs. recreational) marijuana use*.
**Hypothesis 3 (Mediation; Path A** × **Path B):** *Psychological distress will significantly mediate the relationship between perceived social isolation and marijuana use type, such that the effect of isolation on the likelihood of medical use is primarily indirect*.

To test these hypotheses, we analyzed nationally representative data from the 2024 Health Information National Trends Survey (HINTS, Cycle 7) using a cross-sectional survey design. We first characterized sociodemographic and psychosocial differences between medical and recreational users, followed by a survey-weighted structural equation model to evaluate the proposed mediation pathway.

## 2. Materials and Methods

### 2.1. Study Design and Data

Data for this study were drawn from the 2024 HINTS, cycle 7, a cross-sectional survey that provides nationally representative information on adults aged 18 and older living in the U.S., including all 50 states and the District of Columbia [38]. Conducted by the National Cancer Institute, the HINTS tracks patterns of how U.S. residents seek, access, and use health information, as well as assessing related health behaviors in the population. The survey uses a stratified, probability-based sampling framework to obtain a sample reflective of the U.S. population. The sampling frame consists of a comprehensive list of all residential addresses in the U.S., with oversampling of certain populations to ensure adequate representation. Comprehensive documentation of sampling methods, survey administration, and weighting procedures is available in HINTS methodology reports. This project met the criteria for exemption from institutional review board (IRB) review.

#### Inclusion/Exclusion and Analytic Sample

Our inclusion criteria were limited to respondents who completed the 2024 survey and reported valid data for marijuana use. Of the total HINTS 7 respondents (*n* = 7278), we excluded those who: (1) indicated they did not use marijuana (*n* = 5273); (2) provided “not ascertained”, “web partial”, or “multiple responses in error” for the marijuana use type item (*n* = 631); and (3) had missing values for any of the variables included in the analysis (i.e., complete case analysis). The final analytic sample consisted of 1229 U.S. adults who identified as either medical or recreational marijuana users.

### 2.2. Measures

#### 2.2.1. Sociodemographic Variables

Participants provided information on a range of sociodemographic characteristics that were examined in relation to type of marijuana use (medical vs. recreational). Age was categorized into four commonly used life-stage groups: 18–29, 30–44, 45–64, and 65 years and older. Sex was self-reported as male or female. Race/ethnicity was assessed using standard federal classification categories and included non-Hispanic (NH) White, NH Black, Hispanic, NH Asian, and NH Other. Educational attainment was measured as the highest level of education completed and grouped into four categories: less than high school, high school graduate, some college or associate degree, and bachelor’s degree or higher.

#### 2.2.2. Health Related Variables

To account for physical health as a potential confounder, we included the HINTS 2024 self-rated health measure. Respondents were asked the following prompt: “In general, would you say your health is …” with response options on a 5-point Likert scale: excellent, very good, good, fair, and poor [38]. In the analytic model, this was treated as a categorical variable with “excellent” serving as the reference group. Consistent with prior literature using the HINTS data, this measure served as a robust proxy for overall physical health and morbidity. We also included all available chronic conditions available in the HINTS. These were binary variables denoting the presence of: diabetes, high blood pressure, heart conditions, lung disease, and history of cancer.

#### 2.2.3. Marijuana Use Type

Marijuana use type was assessed using a HINTS item asking respondents to indicate the primary reason they used marijuana. Response options included: (1) for medical reasons, (2) for recreational reasons, and (3) for both medical and recreational reasons. We dichotomized the categories into (1) medical (e.g., medical, medical and recreational) and (2) recreational for entry in our analytical models. Several system-coded categories were present to account for nonresponse, skip patterns, and data entry issues. These included values for not ascertained, web partial, filter missing, multiple responses selected in error, commission errors, and an inapplicable category for respondents who indicated that they did not use marijuana. These system-coded values reflected most of the sample and were excluded from analysis because they did not represent valid reports of marijuana use behavior.

#### 2.2.4. Personal Health Questionnaire (PHQ)-4 Score

Depressive and anxiety symptoms were assessed using the Patient Health Questionnaire-4 (PHQ-4), a validated ultra-brief screening measure of psychological distress [39]. The PHQ-4 consists of four items, with two items assessing anxiety symptoms (from the General Anxiety Disorder [GAD]-2) and two items assessing depressive symptoms (from the PHQ-2). Each item asks respondents how often they have been bothered by a symptom over the past two weeks, rated on a 4-point scale: 0 = not at all, 1 = several days, 2 = more than half the days, and 3 = nearly every day. Total scores range from 0 to 12, with higher scores indicating greater overall psychological distress. Standard cut points identify normal (0–2), mild (3–5), moderate (6–8), and severe (9–12) symptom severity. The anxiety and depression subscales each range from 0 to 6. Example items include: “Feeling nervous, anxious, or on edge” (anxiety subscale); “Not being able to stop or control worrying” (anxiety subscale); “Little interest or pleasure in doing things” (depression subscale); “Feeling down, depressed, or hopeless” (depression subscale). The PHQ-4 has demonstrated strong reliability and validity across diverse population groups and is widely used in epidemiologic and clinical research as an efficient indicator of mental health symptom burden. The seminal study establishing this measure reported α = 0.78 in the general population with α = 0.82 for the GAD-2 and α = 0.75 for the PHQ-2 subscales [39]. Results from a systematic review of the PHQ-4 document ranges of α = 0.72 to α = 0.88 for the full scale, with lower ranges for the PHQ-2 (α = 0.65 to α = 0.81) and GAD-2 (α = 0.74 and α = 0.88) [40]. The internal consistency for the PHQ-4 in this sample demonstrated strong reliability at α = 0.90.

#### 2.2.5. Patient-Reported Outcomes Measurement Information System (PROMIS) Social Isolation t-Score

Perceived social isolation was assessed using the PROMIS Social Isolation Short Form, a validated measure designed to capture individuals’ subjective sense of being disconnected from others [41]. The scale includes items that assess feelings of loneliness, exclusion, and lack of companionship, focusing on how often respondents experience social disconnection in their daily lives. Each item is rated on a 5-point Likert scale, ranging from 1 = never to 5 = always, with higher raw scores reflecting greater perceived social isolation. Raw scores are converted to t-scores, which are standardized to have a mean of 50 and a standard deviation of 10 in the U.S. general population. A t-score above 50 indicates higher-than-average perceived isolation, whereas a score below 50 reflects lower levels of isolation. The t-score metric allows for comparisons across studies and populations using a common normed framework. Example items from the PROMIS Social Isolation item bank include statements such as: “I feel left out”; “I feel that people avoid me”; “I feel isolated from others”; “I feel that people barely know me”. The PROMIS system uses Item Response Theory (IRT)to ensure that the short form maintains strong reliability, precision, and validity, even with a brief set of questions. The PROMIS Social Isolation item bank consistently demonstrates high reliability, with internal consistency coefficients typically ranging from α = 0.90 to α = 0.97 in a seminal study of social health item bank development [41]. Validity is supported by strong correlations with “legacy” instruments (like the SF-36 and UCLA Loneliness Scale) and the measures’ ability to differentiate between healthy populations and clinical samples. Because these tools utilize IRT, they provide precise measurement across a wide range of social health levels while minimizing respondent burden. This measure is widely used in population health and behavioral research to assess subjective social disconnection and its relationship to mental and physical health outcomes including studies using cycle 6 of the HINTS, where internal consistency was reported as α = 0.92 [42]. In this sample, the PROMIS scale showed excellent internal consistency with α = 0.92.

### 2.3. Statistical Analysis

For descriptive analyses, weighted frequencies, proportions, means, and Jackknife standard errors were generated to characterize the sample. We calculated descriptive statistics for the full sample and separately for individuals reporting medical versus recreational marijuana use. We used Rao–Scott chi-square tests to assess weighted group differences for categorical variables while weighted one-way ANOVA tests were used to evaluate differences in continuous measures (i.e., PHQ-4 scores and PROMIS Social Isolation t-scores). Prior to model estimation, we evaluated the assumption of multicollinearity among predictors using Variance Inflation Factors (VIFs); all values were below 2.5, indicating no significant collinearity.

All analyses accounted for the complex survey design of the HINTS 2024 (Cycle 7) to ensure that findings were representative of the U.S. adult population. The HINTS provides final person-level sample weights that adjust for differential probabilities of selection, nonresponse, and known population benchmarks through iterative proportional fitting. These weights were applied to produce unbiased population-level point estimates. To account for the survey’s stratified, two-stage probability sampling design and provide accurate standard errors, variance estimation was conducted using 50 replicate Jackknife weights. Prior to analysis, the survey design was specified in the software to incorporate these replicate weights alongside the final person-level weight.

For the mediation analysis, we utilized a survey-weighted structural equation modeling (SEM) approach. The hypothesized model was executed using the svy prefix and Generalized Structural Equation Modeling (gsem) in Stata 19.5. This framework allowed for the simultaneous estimation of (1) a linear regression (identity link) predicting psychological distress (PHQ-4 score), and (2) a logistic regression (logit link) predicting marijuana use type (medical vs. recreational). Missing data on primary predictors and the mediator were handled via listwise deletion, as the proportion of missingness was minimal (<5%). Finally, the indirect effect of perceived social isolation on marijuana use type through psychological distress was evaluated using the Delta method to determine the statistical significance of the mediated pathway. This method provides a computationally stable alternative to bootstrapping when working with complex survey weights. As standard global fit indices (e.g., CFI, RMSEA) are not applicable to weighted GSEM with non-linear links, model selection was guided by the Akaike Information Criterion (AIC) and Bayesian Information Criterion (BIC). Robustness checks were performed by comparing the primary model to a full model including chronic health conditions, as detailed in the Results. All procedures were conducted using Stata 19.5 with svy features (StataCorp, LLC., College Station, TX, USA).

## 3. Results

### 3.1. Sample Distribution and Prevalence of Marijuana Use by Type

Table 1 presents weighted descriptive characteristics by marijuana use type (medical vs. recreational). Several sociodemographic factors showed significant associations with the primary reason for marijuana use. Sex was significantly associated with use type (*p* = 0.007), with a higher proportion of females identifying as medical users (59.6%) compared to males (44.5%). Education level also demonstrated a significant relationship (*p* = 0.001). Those with a college degree were more likely to report recreational use (60.9%), while those with a high school diploma as their highest attainment were more likely to report medical use (65.3%). While the age group distribution showed a trend toward higher medical use among the 65+ cohort (62.3%), this did not reach statistical significance in the bivariate analysis (*p* = 0.143). There was a significant association between self-rated health and marijuana use type (*p* = 0.050). Respondents who rated their health as “fair” reported the highest frequency of medical use (65.3%). Respondents identifying as medical users reported significantly higher mean levels of psychological distress (PHQ-4 *M* = 4.21, *SE* = 0.27) compared to recreational users (*M* = 2.72, *SE* = 0.27, *p* < 0.001). Social isolation t-scores were also significantly higher among medical users (*M* = 50.56, *SE* = 0.78) than recreational users (*M* = 47.13, *SE* = 0.97, *p* = 0.012).

### 3.2. Mediation of the Association Between Perceived Social Isolation and Marijuana Use Type by Psychological Distress, Adjusting for Sociodemographic Covariates

**Figure 1.**
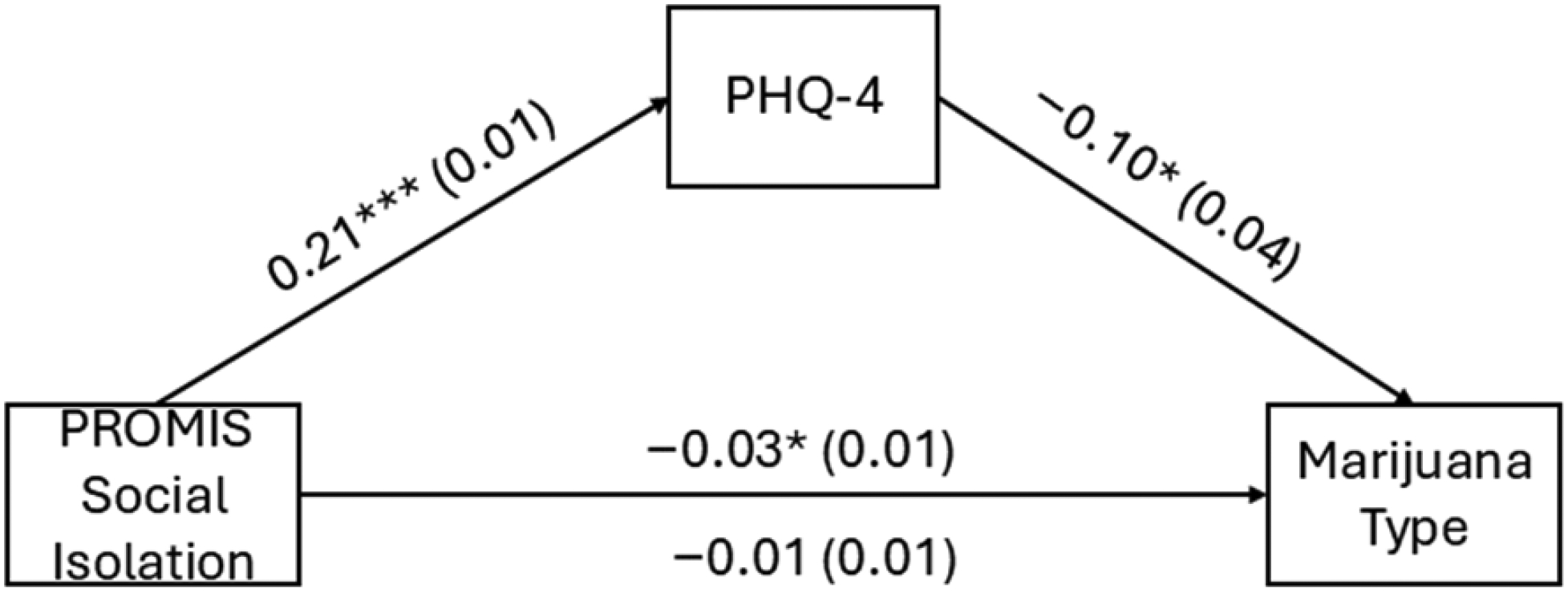
Sociodemographic mediation model with Personal Health Questionnaire (PHQ)-4 score as a Mediator of PROMIS Social Isolation t-score and marijuana use type; * *p* < 0.05, *** *p* < 0.001.

Table 2 presents the survey-weighted path estimates from the mediation model examining whether PHQ-4 psychological distress mediates the association between PROMIS Social Isolation and marijuana use type (medical vs. recreational) and is graphically displayed in Figure 1. Results from the GSEM indicated that greater perceived social isolation was significantly associated with higher psychological distress (*b* = 0.21, *p* < 0.001). In turn, higher psychological distress was associated with lower odds of recreational versus medical marijuana use (*b* = *−*0.10, *p* = 0.016), indicating that individuals with greater distress were more likely to report medical use. The direct association between perceived social isolation and marijuana use type was not statistically significant (*b* = *−*0.01, *p* = 0.527; Table 3). However, there was a significant indirect effect of perceived social isolation on marijuana use type through psychological distress (*b* = *−*0.02, *p* = 0.017), resulting in a significant total effect (*b* = *−*0.03, *p* = 0.006). These findings suggest that psychological distress mediates the relationship between loneliness and marijuana use motivation, such that loneliness increases distress, which in turn is associated with medical rather than recreational marijuana use.

### 3.3. Mediation of the Association Between Perceived Social Isolation and Marijuana Use Type by Psychological Distress, Adjusting for Health Status and Chronic Conditions

**Figure A1.**
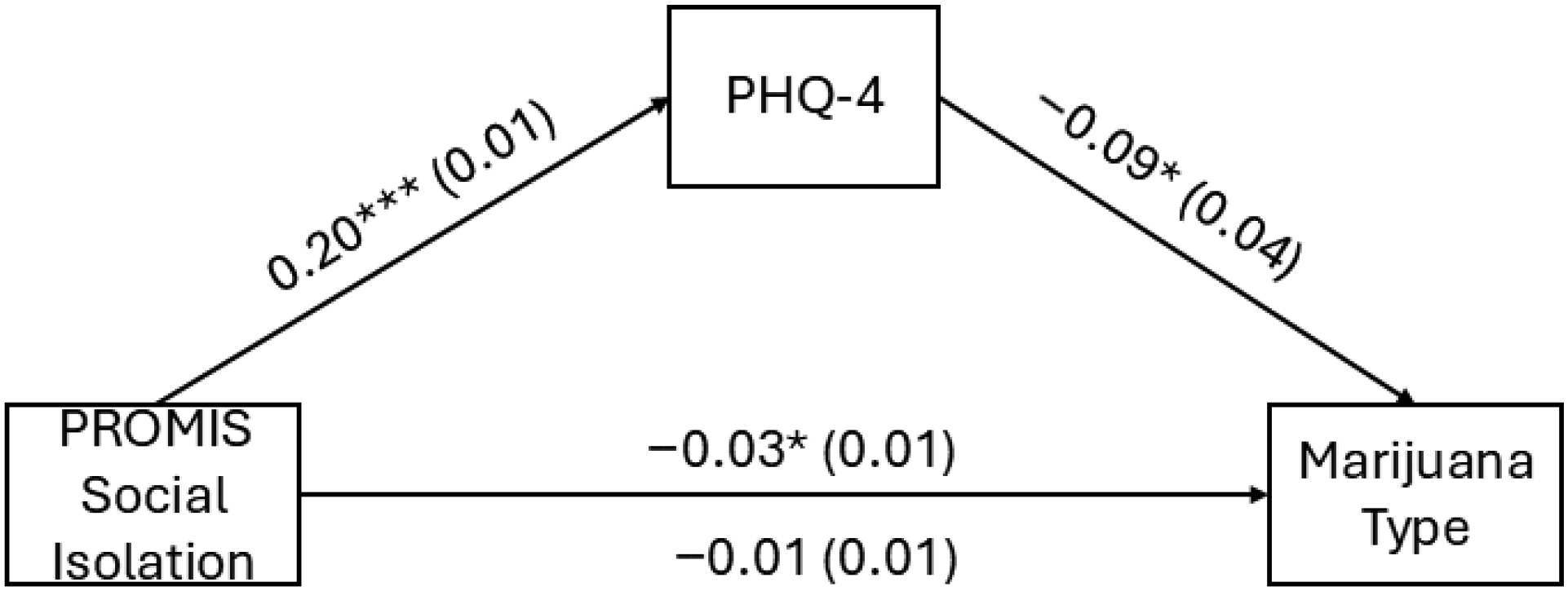
Full mediation model with Personal Health Questionnaire (PHQ)-4 score as a mediator of PROMIS Social Isolation t-score and marijuana use type adjusted for health status and chronic conditions; * *p* < 0.05, *** *p* < 0.001.

In the fully adjusted model reported in Appendix A including self-rated health and chronic physical conditions (Table A1, graphed in Figure A1), greater perceived social isolation remained significantly associated with higher psychological distress (*b* = 0.20, *p* < 0.001). Psychological distress, in turn, was associated with lower odds of recreational versus medical marijuana use (*b* = *−*0.09, *p* = 0.023), indicating that individuals experiencing greater distress were more likely to report medical use. The direct association between loneliness and marijuana use type was not statistically significant (*b* = *−*0.01, *p* = 0.520; Table A2). Mediation analyses indicated a significant indirect effect of perceived social isolation on marijuana use type through psychological distress (*b* = *−*0.02, *p* = 0.024), while the direct effect remained non-significant, resulting in a significant total effect (*b* = *−*0.03, *p* = 0.011). These findings suggest that psychological distress robustly mediates the association between loneliness and marijuana use motivation, independent of physical health status and chronic disease burden. Comparison of model fit indices indicated minimal differences in AIC and BIC between the primary model and the fully adjusted model (Tables 2 and A1), suggesting that adding health status and chronic condition covariates did not substantially improve model fit beyond the more parsimonious specification.

## 4. Discussion

This study examined whether psychological distress mediates the association between perceived social isolation and the likelihood of using marijuana for medical versus recreational purposes among U.S. adults. Consistent with our expectations, the findings indicate that higher perceived social isolation was associated with a greater likelihood of medical (rather than recreational) marijuana use, and this relationship was largely explained by elevated psychological distress. Individuals reporting stronger feelings of social disconnection tended to exhibit higher PHQ-4 distress scores, and in turn, those with greater distress were more likely to use marijuana primarily for medical reasons. After accounting for distress, perceived social isolation no longer had a direct association with marijuana use type, underscoring the central mediating role of psychological distress in this pathway. Secondary findings revealed several demographic distinctions, most notably that older adults and those with poorer self-rated health were more likely to report medical marijuana use, whereas younger adults and males were more likely to report recreational use, but these patterns did not alter the core mediated relationship. Our results largely support the application of the WOT to the context of marijuana use. Under the WOT, individuals are viewed as active agents seeking to maintain a dynamic health equilibrium. Our findings suggest that when this equilibrium is disrupted by social and psychological deficits, individuals may adopt a “medical” framework for cannabis use as a restorative strategy.

Prior cannabis research has largely emphasized prevalence, policy contexts, and health-related outcomes, with comparatively less attention to the motivations underlying different patterns of use. Within this literature, medical marijuana users are typically characterized as older and in poorer physical health than recreational users, reinforcing a predominantly biomedical framing of medical cannabis use [43–46]. However, emerging evidence suggests that motivations for medical use extend beyond symptom management to include emotional regulation, stress relief, and coping with psychological distress [43,44]. Typology-based studies using latent class or profile approaches further demonstrate substantial heterogeneity in cannabis use motivations, with overlapping medical and recreational motives across user groups [47,48]. Despite these advances, psychological distress is often treated as a covariate or outcome rather than as a mechanism shaping use type, even though distress appears more prevalent among individuals using cannabis medically, particularly in policy contexts that expand access [49]. There remains limited understanding of how psychosocial stressors differentiate medical versus recreational marijuana use in nationally representative samples prior to our study.

A growing body of literature links loneliness and perceived social isolation to increased marijuana use, particularly consumption motivated by coping and emotional regulation. As predicted in our first hypothesis, perceived social isolation was significantly and positively associated with psychological distress. This supports the WOT premise that social disconnection is not merely a “social” issue but a systemic health deficit that signals a disruption in the overall well-being equilibrium. Evidence for this association spans qualitative accounts, prospective cohort studies, pandemic-era surveys, and emerging work among older adults, collectively suggesting that social disconnection may function as an important psychosocial stressor driving substance use behaviors [31,50,51]. Studies further indicate that solitary patterns of cannabis use are associated with greater depressive symptoms and elevated risk of cannabis use disorder, underscoring the potential role of isolation-related processes in problematic use trajectories [52,53]. However, much of this literature focuses on any marijuana use or frequency of use rather than distinguishing medical from recreational use, limiting insight into how social isolation may shape use motivations and self-identification as a medical user. Perceived social isolation is rarely disentangled from co-occurring mental health symptom burden, such as depression and anxiety, which are often treated as confounders or mediators rather than analytically distinct constructs. It remains unclear whether social isolation exerts a direct influence on marijuana use type or operates primarily through psychological distress.

Our findings highlight psychological distress as a key mediating mechanism linking perceived social isolation to marijuana-use motivations, offering an important contribution to the literature. Consistent with our second hypothesis, higher levels of psychological distress significantly predicted a greater likelihood of identifying as a medical (vs. recreational) marijuana user. Interpreted through the WOT, this suggests that as the “distress signal” increases, individuals shift their perception of cannabis from an elective or recreational substance to a functional health tool aimed at optimizing their mental health state. Psychological distress is a well-established correlate of cannabis use and is frequently attributed within self-medication frameworks, yet few studies have explicitly tested distress as a mechanism through which social stressors shape the reasons individuals use marijuana. Using a mediation framework, the present study demonstrates that psychological distress mediates the association between perceived social isolation and medical (vs. recreational) marijuana use among U.S. adults. Notably, perceived social isolation was no longer independently associated with use type after accounting for distress, underscoring the central role of mental health in this pathway. By situating social isolation, psychological distress, and marijuana-use motivation within a nationally representative sample, these findings advance biopsychosocial models of cannabis use and suggest that mental health processes may be more proximal drivers of medical use than social disconnection alone.

While the WOT provides a strong explanatory framework, alternative explanations must be considered. First, it is possible that physical health deficits act as a primary driver, causing both social isolation (due to limited mobility, for example) and psychological distress. While our analysis controlled for several chronic conditions, the complex interplay between physical and mental health remains a factor. Additionally, the “medical” label may be influenced by legal access; individuals in states without recreational legality may be more likely to self-identify as “medical” users to justify use, regardless of their distress levels.

The findings of this study carry meaningful implications for both clinical practice and population-level public health efforts aimed at understanding and addressing patterns of marijuana use in the U.S. By demonstrating that psychological distress mediates the association between perceived social isolation and the likelihood of using marijuana for medical rather than recreational purposes, the results highlight the interconnectedness of mental health, social connectedness, and marijuana-use behaviors.

### 4.1. Implications for Psychiatry and Clinical Practice

For psychiatrists and other mental health clinicians including psychologists and social workers, these findings highlight the importance of routinely assessing not only symptom burden but also the social context in which distress occurs. Patients who report using marijuana for medical reasons may be doing so not solely in response to physical health concerns, but as a coping strategy for emotional strain linked to loneliness or perceived social disconnection. In psychiatric settings, this underscores the value of incorporating structured screening for social isolation alongside assessments of mood, anxiety, and substance use. Identifying isolation as a contributing factor may meaningfully alter case formulation and treatment planning.

These findings also suggest that clinicians should carefully explore patients’ motivations for cannabis use during psychiatric evaluation. Distinguishing between use driven primarily by chronic medical conditions versus use motivated by unmanaged psychological distress may inform decisions regarding psychotherapy, pharmacologic treatment, referral to integrated behavioral health services, or social prescribing interventions. For example, individuals presenting with high distress and concurrent medical marijuana use may benefit from evidence-based treatments targeting depression or anxiety, as well as interventions designed to enhance social connectedness, such as social prescribing. Addressing underlying distress and isolation may reduce reliance on cannabis as a self-managed coping mechanism and improve overall psychiatric outcomes.

### 4.2. Implications for Public Mental Health

From a broader public health and psychiatric epidemiology perspective, the study contributes to growing recognition that perceived social isolation functions as a determinant of mental health-related behaviors, including substance use motivations. As cannabis policies continue to evolve nationally, understanding who is using marijuana for medical purposes, including the psychosocial pathways underlying that use, can inform harm reduction strategies, targeted communication efforts, and prevention initiatives within mental health systems.

Public mental health interventions aimed at reducing psychological distress and strengthening community connectedness may have downstream effects on cannabis use patterns. The identification of high-distress subgroups among medical users suggests opportunities for targeted screening and early intervention in outpatient psychiatric clinics, primary care–psychiatry collaborative models, and community mental health settings. Such efforts may be particularly important for individuals who are self-managing distress in the absence of accessible or integrated psychiatric care.

### 4.3. Implications for Research and Policy

For researchers, the findings invite further inquiry into mechanisms linking psychosocial stressors and cannabis use motivations, including potential moderators such as chronic health conditions, social support, psychiatric comorbidity, and state-level regulatory environments. Longitudinal work is especially needed to clarify temporal sequencing and causal pathways relevant to psychiatric treatment planning.

For policymakers and patient communities, the results reinforce the importance of viewing marijuana use through a holistic lens that integrates psychological and social determinants alongside medical need. Efforts to address marijuana use in the U.S., particularly within psychiatric populations, should incorporate systematic mental health assessment and supports for social well-being, ultimately strengthening the precision and impact of both clinical care and public health policy.

### 4.4. Strengths and Limitations

This study offers several notable strengths that enhance confidence in the findings and contribute meaningfully to the literature on social isolation, mental health, and marijuana use. First, the use of a large, nationally representative dataset allows for population-level inferences that extend beyond localized or clinic-based samples commonly used in cannabis research. By leveraging the complex stratified design and incorporating both final sampling weights and replicate Jackknife weights, the analyses appropriately reflect the demographic distribution of U.S. adults and minimize the risk of biased variance estimates. Second, the study applied a survey-weighted structural equation modeling framework, which is still relatively uncommon in cannabis epidemiology. This analytic approach provides a rigorous test of mediating pathways while maintaining fidelity to the complex sampling structure of the HINTS dataset. Third, the integration of validated, psychometrically robust measures, including the PROMIS Social Isolation t-score and the PHQ-4, ensures a high-quality assessment of the central psychological constructs, improving the conceptual clarity and reproducibility of the study.

Several limitations warrant careful consideration. The cross-sectional design prohibits causal inference, meaning that conclusions about temporal ordering among perceived social isolation, psychological distress, and marijuana use motivations should be interpreted with caution. Although the mediation model aligns with theoretically grounded pathways, the temporal relationships remain untested, and reverse or bidirectional effects are plausible. In addition, while HINTS provides rich behavioral and psychosocial data, the measure of marijuana use relies on a single self-reported item about users’ “primary reason” for use. This may oversimplify the motivations of individuals who use cannabis for multiple or evolving purposes, and it does not account for dosage, frequency, or product type, which are factors that may influence both mental health and functional outcomes. The exclusion of system-coded nonresponse categories, although necessary to identify valid users, limits the analytic sample to a relatively small subgroup and may introduce selection bias if individuals with missing marijuana-use data differ systematically from those who responded. Unmeasured confounding also remains possible, particularly regarding factors such as chronic pain severity, medical diagnoses, social support networks, and state-level cannabis policies, none of which were available in the dataset but could influence both distress and medical versus recreational use.

To mitigate these limitations, we incorporated a broad set of sociodemographic and health variables, relied on validated measurement instruments, and used appropriate survey-weighted modeling techniques designed to reduce estimation bias. Still, the results should be understood as associations within the specific population of U.S. adults who reported marijuana use and provided complete data. While generalizability to the broader U.S. population is supported by the national sampling frame, generalizability to clinical subgroups (e.g., individuals with chronic pain, those seeking medical certification) may be more limited. Despite these constraints, the analytic rigor, validated measures, and nationally representative scope strengthen the robustness of the findings and offer a solid foundation for future longitudinal or experimental work to clarify causal mechanisms and refine our understanding of how social isolation and psychological distress shape patterns of marijuana use.

## 5. Conclusions

This study provides novel nationally representative evidence that psychological distress mediates the relationship between perceived social isolation and the likelihood of using marijuana for medical rather than recreational purposes among U.S. adults. While greater social isolation was associated with medical marijuana use, this association operated primarily through elevated psychological distress, and social isolation no longer independently predicted use type after accounting for distress. These findings move beyond descriptive distinctions between medical and recreational users by clarifying a key psychosocial mechanism underlying marijuana-use motivations.

By integrating social isolation, mental health, and cannabis-use motivation within a biopsychosocial framework, the study highlights that medical marijuana use may reflect efforts to cope with emotional distress in addition to physical symptoms. These results underscore the importance of incorporating mental health and social connectedness into clinical assessments and public health approaches addressing cannabis use. Future longitudinal research is needed to establish temporal pathways and to examine how changes in social and psychological contexts shape marijuana-use motivations as cannabis policies and norms continue to evolve.

## Figures and Tables

**Table 1.** Sample characteristics and bivariate comparison by marijuana use type (medical vs. recreational).

	Marijuana Use Type			
		Medical	Recreational	
	*n*	% or *M* (*SE*)	% or *M* (*SE*)	*p*-Value
Age group				0.143
18–29	202	43.8	56.2	
30–44	360	51.2	48.8	
45–64	393	53.9	46.1	
65+	274	62.3	37.7	
Sex				0.007
Female	729	59.6	40.4	
Male	500	44.5	55.5	
Race/ethnicity				0.243
NH White	709	51.8	48.2	
NH Black	184	45.7	54.3	
Hispanic	231	57.4	42.6	
NH Asian	38	19.0	81.0	
NH Other	67	54.9	45.1	
Education level				0.001
Less than high school	65	44.7	55.3	
High school	173	65.3	34.7	
Some college	395	55.1	44.9	
College	596	39.1	60.9	
Health Status				0.050
Excellent	118	41.2	58.8	
Very good	380	45.0	55.0	
Good	452	52.7	47.3	
Fair	229	65.3	34.7	
Poor	50	58.8	41.2	
Diabetes				0.574
Yes	207	48.7	51.3	
No	1022	52.0	48.0	
High Blood Pressure				0.241
Yes	436	56.1	43.9	
No	793	49.5	50.5	
Heart Condition				0.530
Yes	103	57.2	42.8	
No	1126	51.0	49.0	
Lung Disease				0.082
Yes	202	60.9	39.2	
No	1027	49.8	50.2	
History of cancer				0.364
Yes	175	57.3	42.7	
No	1054	51.0	49.0	
PHQ-4	1336	4.16 (0.27)	2.78 (0.26)	0.001
PROMIS Social Isolation t-score	1335	50.40 (0.78)	47.17 (0.93)	0.017

Note. M = mean; SE = Jackknife standard error; NH = non-Hispanic; PHQ-4 = Personal Health Questionnaire-4; PROMIS = Patient-Reported Outcomes Measurement Information System.

**Table 2.** Path estimates from the survey-weighted sociodemographic mediation model predicting Personal Health Questionnaire (PHQ)-4 score and marijuana use type (medical vs. recreational).

Predictor	PHQ-4 *b* (*SE*)	*p*-Value	95% CI	Marijuana Use Type *b* (*SE*)	*p*-Value	95% CI
Constant	−6.43 (0.79)	<0.001	(−7.98, −4.89)	1.87 (0.80)	0.019	(0.30, 3.43)
Age group (ref. = 18–29)						
30–44	−0.18 (0.35)	0.615	(−0.87, 0.51)	−0.61 (0.33)	0.061	(−1.25, 0.03)
45–64	−0.50 (0.36)	0.170	(−1.20, 0.21)	−0.72 (0.33)	0.029	(−1.37, −0.07)
65+	−0.63 (0.38)	0.099	(−1.39, 0.12)	−1.26 (0.37)	0.001	(−1.99, −0.53)
Sex (ref. = Female)						
Male	0.80 (0.26)	0.002	(0.30, 1.30)	−0.56 (0.22)	0.011	(−0.99, −0.13)
Race/ethnicity (ref. = NH White)						
NH Black	−0.29 (0.30)	0.333	(−0.87, 0.30)	0.47 (0.30)	0.115	(−0.11, 1.05)
Hispanic	0.50 (0.33)	0.129	(−0.14, 1.14)	−0.21 (0.30)	0.485	(−0.80, 0.38)
NH Asian	−0.90 (0.47)	0.055	(−1.82, 0.02)	1.04 (0.54)	0.053	(−0.01, 2.10)
NH Other	1.56 (0.79)	0.048	(0.01, 3.10)	0.08 (0.43)	0.845	(−0.80, 0.38)
Education level (ref. = less than high school)						
High school	−0.24 (0.59)	0.692	(−1.40, 0.93)	−0.96 (0.47)	0.041	(−1.87, −0.04)
Some college	−0.45 (0.57)	0.428	(−1.57, 0.67)	−0.47 (0.41)	0.250	(−1.27, 0.33)
College	−0.95 (0.55)	0.084	(−2.02, 0.13)	0.00 (0.40)	0.995	(−0.78, 0.79)
PHQ-4	1.56 (0.79)	0.048	(0.01, 3.10)	0.08 (0.43)	0.845	(−0.80, 0.38)
PROMIS Social Isolation t score Model Fit Statistics						
AIC	3.1 × 10^8^					
BIC	3.1 × 10^8^					

Note. SE = Jackknife standard error; CI = confidence interval; NH = non-Hispanic; PHQ-4 = Personal Health Questionnaire-4; PROMIS = Patient-Reported Outcomes Measurement Information System; AIC = Akaike Information Criterion; BIC = Bayesian Information Criterion.

**Table 3.** Mediation effect estimates of the sociodemographic model.

	b (*SE*)	*p*-Value	95% CI
Indirect path	−0.02 (0.01)	0.017	(−0.04, 0.00)
Direct path	−0.01 (0.01)	0.527	(−0.04, 0.02)
Total	−0.03 (0.01)	0.006	(−0.05, 0.01)

Note. SE = Jackknife standard error; CI = confidence interval.

## Data Availability

Data are freely and publicly available at https://hints.cancer.gov/ (accessed on 1 October 2025).

## Appendix A

**Table A1.** Path estimates from the survey-weighted mediation model predicting Personal Health Questionnaire (PHQ)-4 score and marijuana use type (medical vs. recreational), adjusted for health status and chronic conditions.

Predictor	PHQ-4 *b* (*SE*)	*p*-Value	95% CI	Marijuana Use Type *b* (*SE*)	*p*-Value	95% CI
Constant	−4.80 (1.60)	0.003	(−7.94, −1.67)	2.69 (1.46)	0.065	(−0.17, 5.55)
Age group (ref. = 18–29)						
30–44	−0.36 (0.35)	0.300	(−1.04, 0.32)	−0.64 (0.32)	0.047	(−1.27, −0.01)
45–64	−0.77 (0.35)	0.026	(−1.45, −0.09)	−0.76 (0.33)	0.020	(−1.40, −0.12)
65+	−1.06 (0.37)	0.004	(−1.80, −0.33)	−1.35 (0.39)	0.001	(−2.11, −0.59)
Sex (ref. = Female)						
Male	0.73 (0.25)	0.004	(0.23, 1.22)	−0.52 (0.22)	0.017	(−0.96, −0.09)
Race/ethnicity (ref. = NH White)						
NH Black	−0.36 (0.31)	0.245	(−0.97, 0.25)	0.50 (0.30)	0.102	(−0.10, 1.09)
Hispanic	0.41 (0.33)	0.224	(−0.25, 1.06)	−0.19 (0.30)	0.534	(−0.77, 0.40)
NH Asian	−1.03 (0.41)	0.012	(−1.84, −0.22)	1.02 (0.53)	0.052	(−0.01, 2.05)
NH Other	1.76 (0.80)	0.028	(0.19, 3.33)	0.03 (0.45)	0.948	(−0.86, 0.91)
Education level (ref. = less than high school)						
High school	−0.12 (0.59)	0.837	(−1.27, 1.03)	−1.01 (0.46)	0.029	(−1.91, −0.10)
Some college	−0.37 (0.35)	0.513	(−1.48, 0.74)	−0.57 (0.41)	0.169	(−1.37, 0.24)
College	−0.67 (0.54)	0.216	(−1.73, 0.39)	−0.12 (0.41)	0.777	(−0.92, 0.69)
Health Status (ref. = Excellent)						
Very good	−0.37 (0.35)	0.279	(−1.05, 0.30)	0.23 (0.41)	0.576	(−0.57, 1.03)
Good	0.25 (0.36)	0.488	(−0.46, 0.96)	0.05 (0.40)	0.902	(−0.73, 0.82)
Fair	0.43 (0.54)	0.423	(−0.62, 1.48)	−0.35 (0.45)	0.430	(−1.23, 0.53)
Poor	2.65 (0.64)	<0.001	(1.40, 3.90)	0.21 (0.70)	0.762	(−1.15, 1.57)
Diabetes	0.49 (0.33)	0.136	(−0.15, 1.14)	−0.49 (0.32)	0.118	(−1.11, 0.12)
High Blood Pressure	−0.45 (0.30)	0.131	(−1.03, 0.13)	0.09 (0.26)	0.717	(−0.41, 0.60)
Heart Condition	0.09 (0.48)	0.852	(−0.85, 1.03)	−0.13 (0.42)	0.757	(−0.94, 0.69)
Lung Disease	−0.24 (0.37)	0.514	(−0.97, 0.49)	0.19 (0.33)	0.569	(−0.46, 0.84)
History of Cancer	−0.52 (0.39)	0.174	(−1.28, 0.23)	−0.07 (0.33)	0.832	(−0.72, 0.58)
PHQ-4	-	-	-	−0.09 (0.04)	0.023	(−0.03, 0.02)
PROMIS Social Isolation t score	0.20 (0.01)	<0.001	(0.18, 0.22)	−0.01 (0.01)	0.522	(−0.03, 0.02)
Model Fit Statistics	−0.37 (0.35)	0.279	(−1.05, 0.30)	0.23 (0.41)	0.576	(−0.57, 1.03)
AIC	3.0 × 10^8^					
BIC	3.0 × 10^8^					

Note. SE = Jackknife standard error; CI = confidence interval; NH = non-Hispanic; PHQ-4 = Personal Health Questionnaire-4; PROMIS = Patient-Reported Outcomes Measurement Information System; AIC = Akaike Information Criterion; BIC = Bayesian Information Criterion.

**Table A2.** Mediation effect estimates of the full model adjusted for health status and chronic conditions.

	*b* (*SE*)	*p*-Value	95% CI
Indirect path	−0.02 (0.01)	0.024	(−0.35, −0.00)
Direct path	−0.01 (0.01)	0.522	(−0.03, 0.02)
Total	−0.03 (0.01)	0.011	(−0.05, −0.01)

Note. SE = Jackknife standard error; CI = confidence interval.

## Abbreviations

The following abbreviations are used in this manuscript:

PHQ: Personal Health Questionnaire
PROMIS: Patient-Reported Outcomes Measurement Information System
WOT: Well-being Optimization Theory
